# Cholesterol-crystal embolism presenting with delayed graft function and impaired long-term function in renal transplant recipients: two case reports

**DOI:** 10.1186/1752-1947-3-6839

**Published:** 2009-03-26

**Authors:** Rainer U Pliquett, Aida Asbe-Vollkopf, Ernst H Scheuermann, Elisabeth Gröne, Michael Probst, Helmut Geiger, Ingeborg A Hauser

**Affiliations:** 1 Department of Nephrology, III. Medizinische Klinik, Klinikum der J.W.Goethe Universität, Frankurt/M., Germany; 2 Department of Cellular and Molecular Pathology, Deutsches Krebsforschungszentrum (DKFZ), Heidelberg, Germany; 3 Department of Urology, Klinikum der J.W. Goethe Universität, Frankfurt/M., Germany

## Abstract

**Introduction:**

Impaired renal function and/or pre-existing atherosclerosis in the deceased donor increase the risk of delayed graft function and impaired long-term renal function in kidney transplant recipients.

**Case presentation:**

We report delayed graft function occurring simultaneously in two kidney transplant recipients, aged 57-years-old and 39-years-old, who received renal allografts from the same deceased donor. The 62-year-old donor died of cardiac arrest during an asthmatic state. Renal-allograft biopsies performed in both kidney recipients because of delayed graft function revealed cholesterol-crystal embolism. An empiric statin therapy in addition to low-dose acetylsalicylic acid was initiated. After 10 and 6 hemodialysis sessions every 48 hours, respectively, both renal allografts started to function. Glomerular filtration rates at discharge were 26 ml/min/1.73m^2^ and 23.9 ml/min/1.73m^2^, and remained stable in follow-up examinations. Possible donor and surgical procedure-dependent causes for cholesterol-crystal embolism are discussed.

**Conclusion:**

Cholesterol-crystal embolism should be considered as a cause for delayed graft function and long-term impaired renal allograft function, especially in the older donor population.

## Introduction

Due to organ shortage, the acceptance of older and marginal donors has become necessary. Impaired renal function and/or pre-existing atherosclerosis or prevalent cardiovascular risk factors in the deceased donor increase the risk for delayed graft function (DGF) and impaired long-term renal function in the kidney recipient [1]. We report two kidney recipients with DGF and impaired long-term renal function who received renal allografts from the same donor. In both cases, renal transplant biopsies were performed on day 13 post-transplant revealing cholesterol-crystal embolism (CCE).

## Case presentation

**Figure 1:**
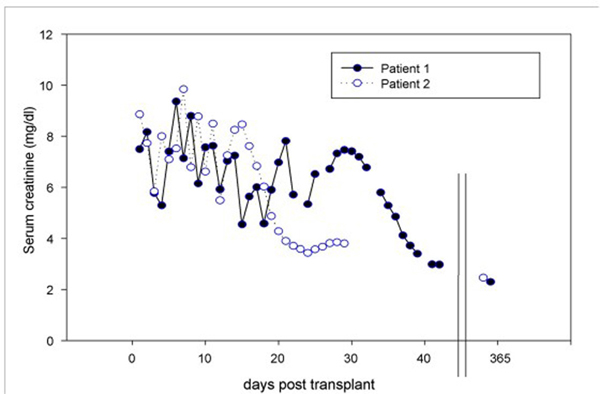
**Renal allograft dysfunction (serum creatinine including the 1-year follow-up) following cholesterol-crystal embolism in two kidney recipients from the same deceased donor**.

Two men, both end-stage renal disease (ESRD) patients, aged 57 years and 39 years respectively, received renal transplants from a deceased donor. Class I and class II human-leukocyte-antigen (HLA) antibodies were positive in one patient pre-transplant. However, the HLAs of the renal allograft were different from the patient's HLA antibodies. In addition, there were two HLA mismatches in each patient. DGF occurred in both patients, lasting for 14 and 21 days, respectively, necessitating 6 and 10 hemodialysis sessions. Once renal function recovered, glomerular filtration rate (GFR) leveled off at 26 ml/min/1.73 m^2^ and 23.9 ml/min/1.73m^2^ by discharge, and remained stable at a reduced level in follow-up examinations (Figure 1).

Both kidney recipients had arterial hypertension as comorbidity. Underlying kidney diseases were Alport syndrome and chronic glomerulonephritis. Both patients had been on chronic, intermittent hemodialysis (CIHD), for 7 and 8.5 years respectively, before the current kidney transplantation. One patient had previously received a deceased-donor renal allograft that had failed after 14 years, so he had resumed CIHD.

The kidney donor was a 62-year-old man with hypoxic brain damage after cardiac arrest for 10 minutes during an acute asthma attack. In addition, he had a history of smoking and needed insulin therapy while in the intensive care unit. Based on the donor's entry laboratory data at the intensive care unit, the estimated GFR (6-variable equation [2]) was 42.4 ml/min/1.73 m^2^. At the time of organ recovery, the renal arteries of the donor were classified as atherosclerotic.

After successful implantation of the renal allografts using an arterial end-to-side anastomosis of the renal artery to the external iliac artery, there was no primary renal-allograft function in both kidney recipients. However, postoperative Doppler ultrasound examinations repetitively showed a homogeneous perfusion in both renal allografts. Resistance indices were between 0.67 and 0.76 (within the normal range).

During transplantation, the warm kidney ischemia period was 46 and 65 minutes, and cold kidney ischemia lasted for 11 hours 40 minutes and 19 hours 6 minutes. Both patients received quadruple immunosuppression consisting of a calcineurin inhibitor, prednisone and mycophenolate mofetil in addition to induction therapy with an interleukin-2 receptor antagonist.

Within 48 hours after transplant surgery, a revision surgery was necessary in one case due to a bleeding complication. The patient received a total of 6 units of packed erythrocytes and remained in a stable condition. The other patient experienced a cytomegalovirus (CMV) reactivation 18 days post-transplant that was successfully treated with ganciclovir for 14 days followed by CMV prophylaxis with valganciclovir. Arterial hypertension worsened in both patients when immunosuppressive therapy was begun. In both patients, repeat physical examination did not reveal evidence of livedo racemosa or purple toes, both indicators of systemic CCE.

Besides laboratory parameters indicative of DGF, other laboratory parameters including C-reactive protein, lactate dehydrogenase and leukocyte count were not found to be elevated. Specifically, there was no increased eosinophil count.

**Figure 2:**
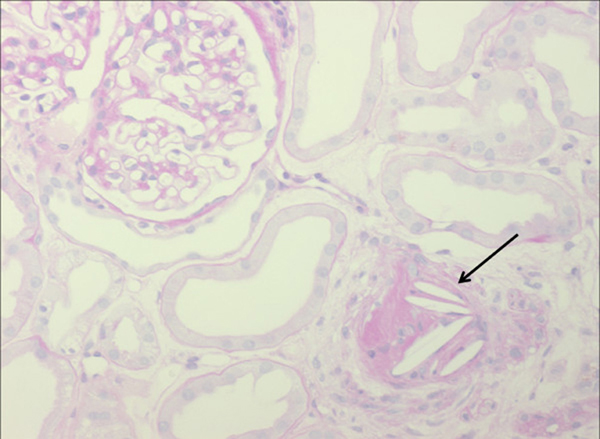
**Renal allograft biopsy of patient 1 shows cholesterol crystals (arrow) in arterioles and interlobular arteries of the renal allograft (Periodic acid Schiff stain, 200×, 3µm)**. Cholesterol clefts are surrounded by macrophages and lymphocytes. Lumina of preglomerular vessels are occluded. Glomeruli are regular. Tubules are characterized by pseudo-dilatation with flattened epithelia with tiny brush border (proximal tubules) and irregular vacuolization (distal tubules).

**Figure 3:**
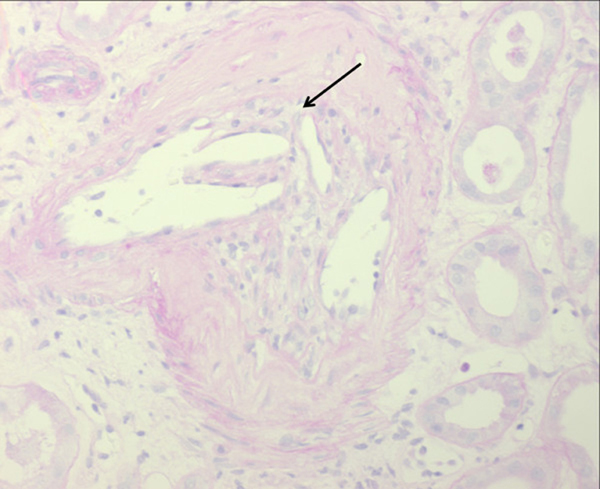
**Renal allograft biopsy of patient 2 shows cholesterol crystals (arrow) in arterioles and interlobular arteries of a renal allograft from the same deceased donor (Periodic acid Schiff stain, 400×, 3µm)**. Cholesterol clefts are surrounded by macrophages and lymphocytes. Lumina of preglomerular vessels are occluded. Glomeruli are regular. Tubules are characterized by pseudo-dilatation with flattened epithelia with tiny brush border (proximal tubules) and irregular vacuolization (distal tubules).

Renal-transplant biopsies performed in both patients on day 13 (34 and 25 glomeruli, Figure 2), and in one patient on day 48 post-transplant (22 glomeruli, Figure 3) ruled out renal-allograft rejection. Moreover, there was no detection of polyomavirus nephropathy or cytomegalovirus antigen in either renal transplant. Besides signs of moderate tubular injury, the histology of the bioptic specimen revealed CCE in arterioles of both renal allografts. Both renal allografts showed moderate to severe nephrosclerosis with narrowing of the arterioles. However, there was only one scarred glomerulus in each biopsy. Subendothelial complement deposits (C1q) were seen in vessels with cholesterol crystals. The biopsies were negative for peritubular C4d or glomerular C3 complement. No baseline renal-transplant biopsy was performed before implantation. The one follow-up renal biopsy showed a picture similar to the index biopsy, however, cholesterol crystals were not detected anymore, and nephrosclerosis was more pronounced.

Fluvastatin, a statin with a good safety profile [3] due to metabolism via the liver cytochrome P450 2C9 isoenzyme, was started in both patients in an attempt to improve endothelial function. To rule out adverse side effects, creatinine kinase was checked regularly. An empiric therapy with acetylsalicylic acid was introduced to counteract the thrombogenic "foreign-body" reaction of cholesterol crystals. Prednisone therapy was given as immunosuppression, and a potential therapeutic role for CCE has been proposed [4].

## Discussion

DGF and impaired long-term allograft function were found to be associated with histologically proven CCE in two renal allograft recipients from the same deceased donor. However, a postoperative bleeding complication in one patient, a prolonged cold and warm ischemia period, and overall allograft quality from a donor fulfilling extended donor criteria may have led to the occurrence of DGF even without CCE. CCE mechanisms for renal allograft failure including microvascular and segmental-artery ischemia may have synergistically compounded with other prevalent risk factors for DGF resulting in irreversible kidney injury as an explanation for the reduced long-term renal-allograft function seen in both recipients.

The importance of CCE for acute kidney injury in native kidneys has recently been reviewed emphasizing the paucity of symptoms except for renal dysfunction [5]. For CCE in renal transplant patients, the same mechanisms and risk factors apply as for the general population. Following renal transplantation, CCE of recipient origin may occur at any time after transplantation. In contrast, CCE of donor origin may have occurred either before or during kidney procurement. Cholesterol crystals emanate from the donor's atherosclerotic renal artery that is clamped or from a crushed plaque. In the cases presented here, the donor appears to be the source for CCE because there were no signs of systemic CCE such as livedo racemosa or hypereosinophilia in the recipients. Secondly, both donor kidneys were affected to the same extent and at the same time following renal transplantation. Pre-existing atherosclerosis, resuscitation efforts, anticoagulation at the intensive care unit and/or surgical manipulation during kidney procurement may have promoted CCE in the kidney donor. There was no indication of repetitive CCE in the one follow-up renal-transplant biopsy.

In the few known cases of CCE to renal transplants, the ratio of recipient versus donor origin of CCE may approximate 3:1 [6]. However, donor-related CCE occurring shortly before or during renal transplantation may go undetected because biopsies of the allograft before implantation or in early phases of DGF are not regularly performed [7,8]. Even if biopsies are performed, the diagnosis of CCE may be missed by nephropathologists as the cholesterol content may be diminished due to processing of the bioptic specimen. As outlined above, other reasons like surgical stress, prolonged cold and warm ischemia periods as well as surgery-related complications may have partly explained DGF in the two kidney recipients. In addition, the renal transplant biopsy proven CCE may have contributed to the DGF in both kidney recipients. CCE may become more prevalent as a differential diagnosis for DGF with older and/or comorbid kidney donors. Moreover, CCE may be one reason for impaired long-term renal allograft function.

In the general population, a longitudinal observational study of proven cases demonstrated that 87% had one or more precipitating risk factors such as angiography. Twenty-four to 33% of patients with renal cholesterol-crystal embolism develop ESRD [9,10]. In the kidneys, cholesterol crystals lead to thrombosis and inflammation due to foreign body reaction thereby altering renal function [7]. Given the detrimental effect of CCE on renal survival in the general population, preventive measures in the donor such as cautious anticoagulation should be taken into consideration. For secondary prevention, statin therapy may mediate plaque stabilization at the sites of origin of CCE. In addition, there is a rationale for acetylsalicylic acid to attenuate platelet activation via cyclooxygenase-dependent pathways, or, alternatively, for steroid therapy as anti-inflammatory treatment strategy [4,11]. Currently, there are no results of randomized clinical trials for the treatment of CCE. Concerning CCE in the renal transplant population, therapeutic recommendations depend on the source of CCE (kidney recipient versus kidney donor) and largely rely on expert opinion [12].

## Conclusions

CCE should be considered as a cause for DGF and poor long-term graft function. Registry studies and/or protocol renal-transplant biopsies may further clarify the prevalence of cholesterol embolism and help investigate treatment strategies. In addition, identification of donors at risk and the prevention of CCE in the donor should be the goal.

## Consent

Written informed consent was obtained from both patients for publication of this case report and any accompanying images. A copy of the written consent is available for review by the Editor-in-Chief of this journal.

## Competing interests

The authors declare that they have no competing interests.

## Authors' contributions

RUP participated in the patients care and drafted the manuscript. AAV participated in the patients' care and gave critical input to the case discussion. EHS substantially contributed to the interpretation of data, literature review, and gave approval as head of transplantation unit. HG provided clinical insights and final approval for the manuscript as the head of department. MP contributed in patient care in all surgical aspects and revised critically respective sections in the manuscript. EG gave substantial input on histology-related issues. IAH participated in patient´s care as attending physician and revised the manuscript. All authors read and approved the manuscript.
